# The interaction between farming/rural environment and *TLR2, TLR4, TLR6 and CD14* genetic polymorphisms in relation to early- and late-onset asthma

**DOI:** 10.1038/srep43681

**Published:** 2017-03-06

**Authors:** Melisa Y. Z. Lau, Shyamali C. Dharmage, John A. Burgess, Aung K. Win, Adrian J. Lowe, Caroline Lodge, Jennifer Perret, Jennie Hui, Paul S. Thomas, Stephen Morrison, Graham G. Giles, John Hopper, Michael J. Abramson, E. Haydn Walters, Melanie C. Matheson

**Affiliations:** 1 Allergy and Lung Health Unit, Centre for Epidemiology and Biostatistics, Melbourne School of Population and Global Health, the University of Melbourne, Victoria, Australia; 2 Murdoch Childrens Research Institute, Melbourne, Victoria, Australia; 3 School of Population Health, the University of Western Australia, Perth, Australia; 4 Inflammation and Infection Research Centre, University of New South Wales, Australia; 5 Department of Medicine, the University of Queensland, Queensland, Australia; 6 Cancer Epidemiology Centre, Cancer Council Victoria, Victoria, Australia; 7 School of Public Health & Preventive Medicine, Monash University, Melbourne, Victoria, Australia; 8 School of Medicine, University of Tasmania, Tasmania, Australia

## Abstract

Asthma phenotypes based on age-of-onset may be differently influenced by the interaction between variation in toll-like receptor (*TLR*)/*CD14* genes and environmental microbes. We examined the associations between single-nucleotide polymorphisms (SNP) in the *TLR*/*CD14* genes and asthma, and their interaction with proxies of microbial exposure (childhood farm exposure and childhood rural environment). Ten SNPs in four genes (*TLR2, TLR4, TLR6, CD14*) were genotyped for 1,116 participants from the Tasmanian Longitudinal Health Study (TAHS). Using prospectively collected information, asthma was classified as never, early- (before 13 years) or late-onset (after 13 years). Information on childhood farm exposure/childhood rural environment was collected at baseline. Those with early-onset asthma were more likely to be males, had a family history of allergy and a personal history of childhood atopy. We found significant interaction between *TLR6* SNPs and childhood farm exposure. For those with childhood farm exposure, carriers of the *TLR6*-rs1039559 T-allele (p-interaction = 0.009) and *TLR6*-rs5743810 C-allele (p-interaction = 0.02) were associated with lower risk of early-onset asthma. We suggest the findings to be interpreted as hypothesis-generating as the interaction effect did not withstand correction for multiple testing. In this large, population-based longitudinal study, we found that the risk of early- and late-onset asthma is differently influenced by the interaction between childhood farming exposure and genetic variations.

Farm and rural environment are associated with high load of microbial compounds from gram-negative and gram-positive bacteria and fungi^1^ and is thought to reduce the risk of asthma through modulation of the immune system. High exposure to microbial compounds such as lipopolysaccharide (LPS)/endotoxin, lipopeptide and muramic acid may deviate the Th1/Th2 immune balance by activating the Th1 response that in turn suppresses the Th2 response^2^. However, in a low microbe environment, the immune response is redirected to favour a Th2 response, therefore predisposing the host to allergic diseases^3^. These microbial compounds initiate a response through interaction with innate immunity receptors such as the Toll-like receptors (TLRs) and CD14^4^,^5^.

The TLRs and CD14 receptors are pattern recognition receptors (PRR) found on the surface of macrophages and dendritic cells^6^. The CD14 is also found in a soluble form in the serum and confers LPS specificity to cells lacking CD14 receptors on the surface^7^. Recognition of microbial products by TLRs and CD14 induces production and release of Th1 proinflammatory cytokines such as IL-1 and TNF-α^8^. As TLRs and CD14 were heavily involved in the recognition of microbial compounds, genetic variation of these receptors can have a substantial effect on Th1/Th2 immune balance that directly influence the risk of asthma. Single nucleotide polymorphisms (SNPs) in the *TLRs* and *CD14* genes have been associated with a change in amino acid sequence leading to a change in protein structure^9^, the level of gene expression^4^ and serum IgE levels^10^. Despite evidence of a possible biological mechanism by which genetic variations in these SNPs may influence asthma risk, studies examining the association between the SNPs in the *TLRs* and *CD14* genes and asthma risk have been inconclusive^11^.

Given the complex interaction between *TLR*/*CD14* genes and the environment, studies that have looked at either factor individually have produced inconsistent results. We recently conducted a systematic review of gene-environmental interactions for the *CD14* gene and markers of microbial exposure. We found that high endotoxin exposure in early-life was associated with a lower risk of atopy in children with the CC genotype of the *CD14*-rs2569190 SNP^12^. Our review also identified a clear differential pattern of association based on childhood versus adult disease. The majority of gene-environmental interaction studies have focused on intermediate phenotypes of allergic disease such as IgE level and atopy with few studies in asthma^13^,^14^ and none have explored different phenotypes of asthma based on age. Refinement of asthma phenotype is critical to the examination of gene-environmental interactions as different asthma phenotypes such as those based on age of onset, are known to have distinct characteristics^15^.

Based on their importance in the immune response to microbial exposure we aimed to investigated the gene-environmental interaction between ten SNPs in the *TLR2* (rs4696480, rs1898830, rs3804100), *TLR4* (rs1927911, rs46986790), *TLR6* (rs1039559, rs5743810) and *CD14* (rs2915863, rs5744455, rs2569190) genes and exposure to childhood farm exposure or childhood rural environment on the risk of early-onset asthma or late-onset asthma. The selection of SNPs were based on previous candidate gene studies that had examined the relationship with either asthma, hay fever or atopic sensitisation (Table S1). The selection of CD14 *rs2569190, rs5744455* and *rs2915863* SNPs were based on their relationship with asthma or wheeze, sensitisation or hay fever^12^. The *TLR6* rs5743810 and rs1039559 SNPs was selected because they have been shown to be associated with hay fever and sensitisation in three large GWAS studies^16^,^17^,^18^. The SNPs from the *TLR2* and *TLR4* were selected based on the associations shown with asthma and atopy, and their effect modification by farming exposure in a previous study^19^.

We utilised data from the Tasmanian Longitudinal Health Study (TAHS)^20^, a population-based cohort followed from childhood to adulthood to examine the associations. We investigated if exposure to a farming environment or living in a rural area in childhood would modify the association.

## Results

### Characteristics of the study population

**Figure 1:**
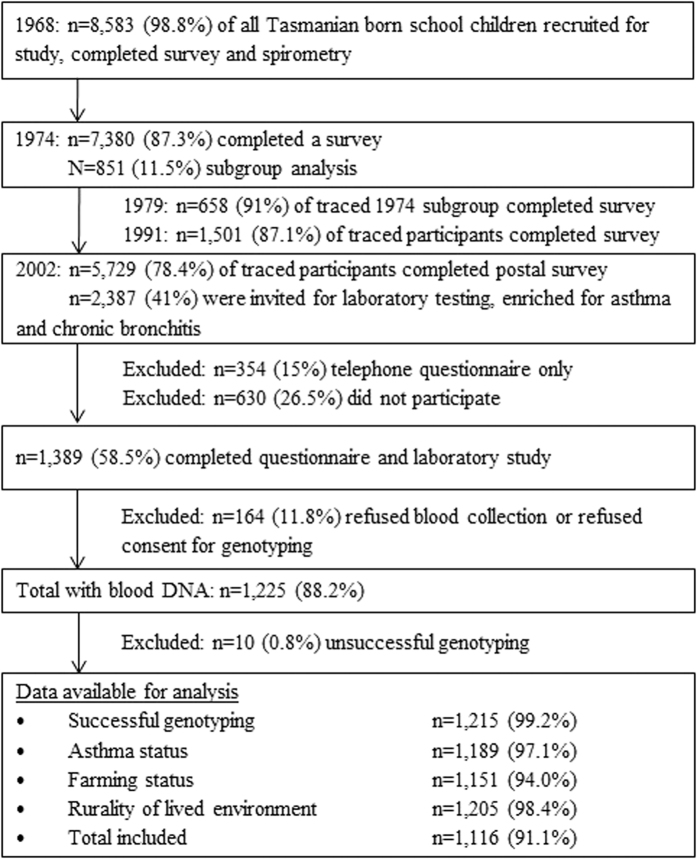
Study design of the TAHS with data available for this study. Adapted from Matheson *et al*. Cohort Profile: The Tasmanian Longitudinal Health Study (TAHS), 2016^20^. The differences in the data available for analysis is due to missing data.

A total of 1,116 participants had both genotyping and data collected asthma and early-life environmental exposures and were included in this analysis (Fig. 1). General characteristics of participants who were included in this study is shown in Table 1. About forty-eight percent of the participants (n = 534) had early-onset asthma, 21.8% (n = 243) had late-onset asthma and 30.4% (n = 339) had never reported asthma over their lifetime. The median age of asthma for early-onset asthma was 3 years (interquartile range [IQR] 1–5) and 30 years (IQR 25–38) for late-onset asthma. Those with early-onset asthma were more likely to be males, had a history of maternal and paternal asthma and/or hay fever and a personal history of hay fever and eczema at age 7 years. Those with late-onset asthma were more likely to have current asthma and were using asthma medication (inhaled corticosteroid) at the 2002 follow-up.

### Association between proxies of microbial exposure and asthma onset

We did not find an association between childhood rural environment and early- nor late-onset asthma (Table 2). There was also no evidence of an association between childhood farm exposure and either early-onset asthma or late-onset asthma.

### Association between *TLR2, TLR4, TLR6* and *CD14* polymorphisms and asthma onset

The characteristics and distributions of the genotypes for each SNP is shown in Table 3. For the genotype frequencies for each of the ten SNPs, there was no marked deviation from that expected under the HWE. The AIC and BIC scores was used to determine the best fitting genetic model for each SNP (Table S3). The association between each SNP and asthma risk was examined however we did not find any association between the SNPs and early-onset or late-onset asthma (Tables 4 and S2).

### Interactions between genes and environment

We investigated whether childhood farm exposure or childhood rural environment modified the association between the *TLR* and *CD14* SNPs and risk of early- and late-onset asthma. There was strong evidence for interactions between the *TLR6*-rs1039559 and *TLR6*-rs5743810 SNPs and childhood farm exposure for early-onset asthma (p = 0.009 and p = 0.02 respectively, Tables 5 and S5). For those with childhood farm exposure, the *TLR6*-rs1039559 T-allele was associated with a markedly reduced risk of early-onset asthma (additive genetic model for the T-allele OR = 0.34, 95%CI 0.16–0.73), but not for those without childhood farm exposure. Again in those with childhood farm exposure having the *TLR6*-rs5743810 C-allele was associated with a lower risk of early-onset asthma (additive genetic model for the C-allele OR = 0.41, 95%CI 0.19–0.86). No interactions were observed for those with late-onset asthma (Tables 5 and S4).

There was no evidence for an interaction between *TLR6* polymorphisms and childhood rural environment for either early- or late-onset asthma (data not shown). Nor was there any evidence for an interaction between any of the SNPs and either childhood farm exposure or childhood rural environment in relation to the risk of early- or late- onset asthma (Table S5).

Reanalyses of the interaction effects in this study did not withstand correction for multiple testing (data not shown). The p-values for the analyses are presented without adjustment and the results should be viewed as hypothesis-generating rather than proof of a causal association.

### Sensitivity analysis for significant interactions

We performed a sensitivity analysis that further refined early-onset asthma phenotypes into early-onset transient asthma and early-onset persistent asthma. Specifically, we examined the interaction between *TLR6* SNPs and childhood farm exposure for these early-onset asthma phenotypes and found comparable findings for both phenotypes. The findings for each groups were comparable to the combined early-onset asthma group (Table S6). Those with the *TLR6*-rs1039559 T-allele with childhood farm exposure had lower risk of early-onset transient and early-onset persistent asthma compared to those without childhood farm exposure. The findings were similar for the *TLR6*-rs5743810 whereby those with the C-allele and childhood farm exposure had reduced risk of early-onset transient asthma and early-onset persistent asthma. We then removed individuals who had asthma before age 5 years and repeated the analysis of our main findings (Table S7). The findings for the interaction between *TLR6* and childhood farm exposure for asthma were strongly comparable to the findings with all participants included.

## Discussion

Using a longitudinal cohort that has been followed from childhood (age 7 years) into adult-life (age 45 years when this clinical study was conducted), we identified an effect modification by farm exposure in childhood on the association between *TLR6* SNPs and asthma phenotypes based on age of onset. Being exposed to a farm environment in childhood was protective against early-onset asthma for those with *TLR6*-rs1039559 T-allele and *TLR6*-rs5743810 C-allele, showing a clear difference in the pattern of interaction for early- and late-onset asthma. We did not observe any association between *TLR2, TLR4* or *CD14* SNPs and asthma onset, nor was there any modification of these relationships by childhood farm exposure/childhood rural environment.

The *TLR6* is located on chromosome 4p14, a region that was consistently associated with atopy in three GWAS^16^,^17^,^18^. Early-onset asthma is more likely to be attributable to atopy^15^ and therefore it is not surprising to observe some evidence for the association between the two *TLR6* SNPs and early-onset asthma but not late-onset asthma in our study. In our data, early-onset asthma were more likely to have a history of atopy in childhood than those with late-onset asthma. Only about 22% of those with early-onset asthma had adult current asthma whereas about 59% of late-onset asthma had adult current asthma by age 44 years. We have also shown previously that early- and late-onset asthma exhibit different clinical characteristics in our study^21^. Our findings were also consistent with several other studies that have also found some evidence of an association between *TLR6*-rs5743810 T-allele and atopic asthma^9^, allergic rhinitis^22^ and allergic sensitisation^23^ in children.

We are aware of only one previous study that examined the interaction between *TLR6* SNPs and proxies of microbial exposure and allergic disease outcomes^23^. In contrast to our finding of a lower risk of asthma in individuals with the *TLR6*-rs1039559 T-allele and early farm exposure, their study found higher IgE levels at age two years in individuals with the *TLR6*-rs1039559 CC-genotype and exposure to two or more older siblings. The inconsistency with our findings may be due to the difference between our proxy measures of microbial exposure (farm exposure and rural environment) and that used by Reijmerink *et al*. (siblings). Reijmerink *et al*. finding with regard to exposure to siblings leading to higher IgE is in contrast to most existing literature.

There is some evidence from *in vitro* studies of a variable response to bacterial cell products with the *TLR6*-rs5743810 SNP. A polymorphism of C to T in the *TLR6*-rs5743810 resulted in a change in amino acid from proline to serine at position 249 in the extracellular domain of the receptor. Although the effect on the structure is still unknown, studies using peripheral blood cells shown that the *TLR6*-rs5743810 C-allele showed greater NF-κB signalling activity in response to stimulation by lipopeptide when compared to the *TLR6*-rs5743810 T-allele^24^. The higher NF-κB signalling in turn resulted in higher *TLR6* mRNA gene expression and higher IFN-γ levels, thereby promoting greater Th1-immunity with a matching reduction of Th2-immunity^9^. This mechanism could explain the observed reduced risk of asthma in those with the *TLR6*-rs5743810 C-allele who were exposed to a farm environment in our study.

We did not find any evidence of modification by childhood farm exposure or childhood rural environment on the relationship between any of the *CD14* SNPs and asthma risk. This is consistent with a case-control study of adults that found no association with the *CD14*-rs2569190 SNPs and asthma for those who lived in the country in childhood^13^. A systematic review of GxE in the *CD14* gene showed consistent evidence of a decreased risk of allergic sensitisation in childhood when exposed to high levels of endotoxin in carriers of the *CD14*-rs2569190 CC-genotype^12^. Importantly this consistent effect was only seen for atopy assessed in childhood, and no consistent effect was observed for outcomes other than allergic sensitisation. Unfortunately we did not have an objective marker of microbial exposure such as endotoxin levels in the current study to fully examine the relationship between *CD14* and microbial exposure and asthma risk.

At present, any effect of the *TLR2* and *TLR4* gene polymorphisms on asthma is still largely undetermined. A recent meta-analysis showed no evidence of association between *TLR4*-rs4986790 and asthma^25^. For *TLR2*, two studies found a reduced risk of asthma in children with A-alleles of the rs4696480 SNP^19^,^26^. Another study found a reduced risk of doctor-diagnosed asthma in adults with a T-allele of the *TLR2*-rs4696480 SNP^27^. Two other studies, however, reported no association between asthma risk and *TLR2*-rs4696480 in children^9^ and adult^28^. We found no evidence of association between *TLR2*-rs4696480 and asthma, and the association was not modified by childhood farm exposure or childhood rural environment. The inconsistency in the findings across studies may be due to variation in the definition of asthma across studies.

Farm exposure is commonly used as a proxy for microbial exposure^1^ and has been consistently reported as protective against atopy and asthma, albeit mainly in cross-sectional studies^29^. We did not find any association between childhood farm exposure and asthma per se in the current analysis. Past studies of childhood farming exposure have used a variety of ways to define “farming exposure” including “growing up on a farm/farm residence”^30^, “physical contact with farm animals in childhood”^31^, “regular consumption of unpasteurised farm milk”^14^ and “parental farming occupation”^32^. Of these, “farm residence” and “parental farming occupation” have been most utilised in epidemiological studies^29^. We used father’s reported occupation when the child started school to define exposure. Since information on father’s occupation was collected prior to parental report of asthma status in the child, any misclassification of true exposure to a “farm environment” is likely to be non-differential. We did not find an association between childhood rural environment and early-onset and late-onset asthma. Classification of childhood rural environment was based on the postcode of the school that the child attended in year 1968, and hence, it may not capture the true effect of microbial exposures.

Strengths of our study include the large cohort of individuals and the prospective data collection enabling us to accurately classify participants into different asthma phenotypes based on age of onset of disease and also to define the proxies of microbial exposure prior to the baseline assessment of asthma. The use of self/parental-reported asthma is a limitation as it is likely to be less reliable than a physician’s diagnosis. However our definitions of self/parent-reported asthma has been validated against a definition that included bronchial hyper-responsiveness^33^.

In conclusion, we have shown for the first time that farming exposure in early-life modified the relationship between polymorphisms in the *TLR6* gene and asthma that started in early life. Our finding may provide some explanation to the lack of consistent evidence in genetic and gene by environment interaction studies especially when asthma phenotypes based on age of onset were not clearly defined. Replication of our finding using longitudinal studies with repeated measurements of objective markers such as endotoxin and distinct phenotypes of asthma are needed to fully explore these relationships. Our work has provided some indication of a possible mechanism in which microbial products may interaction with *TLR* in a well-defined asthma group.

## Methods

### Study design – Tasmanian Longitudinal Health Study (TAHS)

The design of the TAHS, as well as the prevalence of asthma at baseline and follow-up, has been reported elsewhere^20^. In brief, the TAHS commenced in 1968 with recruitment of all school children in Tasmania born in the year 1961 and 8,583 (99% of all eligible subjects) were enrolled. These children (probands) underwent clinical examinations including lung function measurement, and parents completed a respiratory health survey for each child. The parents also completed separate surveys for themselves. Follow-up surveys were completed in 1974, 1981 and 1992 at the ages of 13, 20 and 31 years, respectively.

The 2002 follow-up survey traced 7,380 (87.31%) of the original 1968 cohort to an address^34^ and achieved a response from 5,729 (78.4%) to a postal survey. A subgroup of these respondents, enriched for cases of asthma or cough reported in childhood or adulthood, were invited to participate in a more detailed laboratory study. Of the 2,387 invited, 1,389 (58.5%) took part in a full laboratory visit, 354 (15%) completed a telephone questionnaire or laboratory visit only, and 630 (26.5%) withdrew. This analysis was based on those who had successful genotyping (n = 1,215). Of those who provided their blood sample for genotyping, majority of them (97.6%) were born in Tasmania (93.4%) or other states in Australia (4.2%) and are of Anglo-Celtic ancestry.

### Ethics

This study was conducted in accordance with the amended Declaration of Helsinki and approved by the Human Ethics Review Committees at The Universities of Melbourne (approval number 040375), Tasmania (040375.1) and New South Wales (08094), the Alfred Hospital (1118/04), and Royal Brisbane & Women’s Hospital Health Service District (2006/037). Informed written consent was obtained from all study participants at each follow-up.

### Early- and Late-onset Asthma

Asthma was defined by an affirmative response to the question: “Has he/she at any time in his/her life suffered from attacks of asthma or wheezy breathing?” This question was asked in all follow-up surveys and in the 1968 and 1974 studies the parents answered on behalf of their children, but in 1992 and 2004 the participants answered for themselves. Participants who reported asthma in the 1968 or 1974 surveys were classified as having “Early-onset asthma” and asthma reported in the 1992 or 2004 surveys was classified as “Late-onset asthma”. The reference group was those who did not report asthma at any survey. The cut-off for early onset was set at the age of 13 years which has been consistently used in previous studies^15^ and we have shown some differences in characteristics within the TAHS using this cut-off 21.

### Definitions of Exposure

Two proxy measures of exposure to a farming or rural environment in our study:

“**Childhood farm exposure**” was defined from the father’s occupation obtained from the school medical record completed by the parents before the child commenced primary school when aged 5 years. Occupations were coded using the Australian Standard Classification of Occupations (ASCO) four-digit classification^35^ and codes 1311–1314, 4611–4614, and 9211 were defined as “Farmer”.

“**Childhood rural environment**” was measured by residential remoteness using the postcode of the school that the child attended in 1968 and was based on the Australian Standard Geographical Classification (ASGC)^36^. “Rural environment” was defined as those in groups ‘outer regional Australia’, ‘remote Australia’ and ‘very remote Australia’.

### Definitions of baseline characteristics

‘Maternal asthma and/or hay fever’ and ‘paternal asthma and/or hay fever’ were defined by a parent having asthma and/or hay fever from the 1968 survey. ‘Parental smoking’ was defined as probands with either or both parents who reported a history of smoking in the 1968 survey. Childhood eczema and hay fever was reported by parents at the 1968 survey.

### Genotyping

DNA samples were isolated from whole blood samples obtained from each participant. Genotyping was performed for the following genes: *TLR2* (rs4696480, rs1898830, rs3804100), *TLR4* (rs1927911, rs46986790), *TLR6* (rs1039559, rs5743810) and *CD14* (rs2915863, rs5744455, rs2569190). The selection of SNPs were based on previous candidate gene studies that had examined the relationship with either asthma, hay fever or atopic sensitisation^12^,^16^,^19^,^37^. Genotyping was performed using Taq-Man allelic discrimination 5′nuclease assays (Applied Biosystems, Foster City, CA, USA) according to the manufacturer’s protocol. Fluorescence was measured by an ABI7900HT Fast Real-Time PCR System (Perkin-Elmer, Waltham, MA, USA).

### Sensitivity analysis

To further explore other phenotypes within early-onset asthma, we have categorised early-onset asthma into transient early-onset asthma and persistent early-onset asthma. Individuals with early-onset asthma who also reported having asthma at age 45 years was categorised into early-onset persistent asthma while individuals who did not have asthma at age 45 years was group into early-onset transient asthma. Asthma under 5 years of age may not be true asthma and hence we repeated our main analysis by excluding participants who had their first asthma attack before the age of 5 years.

### Statistical analyses

All analyses were performed using STATA ver13.1 (StataCorp LP, College Station, TX). Deviation from Hardy-Weinberg equilibrium was tested using a Pearson chi-squared test in the reference group (never asthma).

The association between proxies of microbial exposure or SNPs and early-onset/late-onset asthma was assessed using multinomial logistic regression. All analyses were adjusted for sex, maternal and paternal asthma and/or hay fever at baseline, and atopy at age 7 years.

The association between the SNP and asthma outcomes was examined using all four types of genetic models (dominant, recessive, additive and codominant). To determine the best fitting genetic model for the interaction analysis, we compared the Akaike’s information criteria (AIC) and Bayesian information criteria (BIC) for each genetic model for each SNP (Table S3). The model with the lowest AIC and BIC scores was selected and presented in the manuscript. The interaction between SNPs and childhood farm exposure or childhood rural environment was examined using a multinomial logistic regression while adjusting for confounders. The interaction was examined by comparing each model with the interaction term to the same model without an interaction term using a likelihood ratio test. A p-value of less than 0.1 from the likelihood ratio test indicates that the models were different and the estimates for the interaction in each subgroup were presented. The p-value from the interaction odds ratio was used to determine the significance of interaction term. For sensitivity analysis, we have only analysed models with significant interaction. The association was analysed in the same manner using multinomial logistic regression.

## Additional Information

**How to cite this article:** Lau, M. Y. Z. *et al*. The interaction between farming/rural environment and *TLR2, TLR4, TLR6 and CD14* genetic polymorphisms in relation to early- and late-onset asthma. *Sci. Rep.* **7**, 43681; doi: 10.1038/srep43681 (2017).

**Publisher's note:** Springer Nature remains neutral with regard to jurisdictional claims in published maps and institutional affiliations.

## Supplementary Material

Supplementary Information

## Figures and Tables

**Table 1:** General characteristics of the asthma phenotypes.

Baseline characteristics	Never asthma n/N (%)	Early-onset asthma n/N (%)	Late-onset asthma n/N (%)
**Childhood personal factors**			
Sex – Male	174/356 (48.9)	312/579 (53.9)	106/260 (40.8)
Hay fever at 7 years	32/350 (9.1)	172/559 (30.8)	26/256 (10.2)
Eczema at 7 years	25/351 (7.1)	149/565 (26.4)	32/256 (12.5)
**Parental Factors at baseline**			
Maternal asthma and/or hay fever	80/350 (22.9)	215/553 (38.9)	64/256 (25.0)
Paternal asthma and/or hay fever	62/343 (18.1)	197/539 (36.6)	51/248 (20.6)
Parental smoking	219/338 (64.8)	371/543 (68.3)	159/250 (63.6)
Maternal age at birth of proband (years ± s.d.)	27.7 ± 5.8	27.1 ± 5.9	27.2 ± 5.6
Social class			
Managers/administrators	73/337 (21.7)	133/541 (24.6)	54/245 (22.0)
Associate professionals	26/337 (7.7)	52/541 (9.6)	17/245 (6.9)
Tradesperson/advance clerical	90/337 (26.7)	163/541 (30.1)	85/245 (34.7)
Intermediate production/sales/clerical	104/337 (30.9)	139/541 (25.7)	65/245 (26.5)
Labourer/house person	44/337 (13.1)	54/541 (9.9)	24/245 (9.8)
**Adult personal factors**			
Current asthma at 45 years	–	132/575 (22.9)	148/258 (57.4)
Medication – current use of inhaled corticosteroid	–	111/575 (19.3)	85/258 (33.0)
Any sensitisation at 45years*	138/349 (39.5)	364/563 (64.7)	156/258 (60.5)
Highest education			
Grade 9 or below	17/353 (4.8)	33/574 (5.7)	18/257 (7.0)
Grade 10 or 11	116/353 (32.9)	146/574 (25.4)	78/257 (30.4)
Grade 12 or equivalent	35/353 (9.9)	57/574 (9.9)	26/257 (10.1)
Trades/apprenticeship	55/353 (15.6)	97/574 (16.9)	33/257 (12.8)
Certificate of diploma	63/353 (17.9)	124/574 (21.6)	58/257 (22.6)
University degree	53/353 (15.0)	81/574 (14.1)	28/257 (10.9)
Higher university degree	14/353 (4.0)	36/574 (6.3)	16/257 (6.2)

^*^Positive skin prick test to one or more of the following allergens using standard techniques: *Dermatophagoides pteronyssinus* (house dust mite), cat pelt, *Homodendrum, Alternaria tenuis, Penicillium* mix, *Aspergillus fumigatus*, and mixed grasses^20^.

**Table 2:** The association between proxies of microbial exposures and asthma onset.

Proxies of microbial exposures	Never asthma n/N (%)	Early-onset asthma n/N (%)	Late-onset asthma n/N (%)	Early-onset asthma vs Never asthma		Late-onset asthma vs Never asthma	
				OR (95% CI)	p-value for OR	OR (95% CI)	p-value for OR
Childhood rural environment	125/354 (35.3)	204/572 (35.7)	98/257 (38.1)	0.94 (0.69–1.27)	0.67	1.04 (0.72–1.50)	0.67
Childhood farm exposure	26/340 (7.7)	52/545 (9.5)	23/247 (9.3)	1.21 (0.71–2.07)	0.48	1.23 (0.67–2.25)	0.50

^*^All analyses were adjusted for sex, maternal and paternal history of asthma and/or hay fever, parental smoking, maternal age, and childhood history of eczema and/or hay fever.

**Table 3:** The location, genotype distribution, Hardy-Weinberg equilibrium and minor allele frequencies of the SNPs included in this study.

Gene	SNP	Position	Location in gene	Genotype distribution^a^			HWE p-value	MAF		
				No asthma	Early-onset asthma	Late-onset asthma		No asthma	Early-onset asthma	Late-onset asthma
*TLR2*	rs4696480	−16934 A > T	Intron	98/173/85	148/298/125	64/131/63	0.60	0.482	0.479	0.504
	rs1898830	−15607 A > G	Intron	163/145/49	268/242/62	113/107/38	0.08	0.340	0.319	0.355
	rs3804100	+1349 T > C	Coding region	312/42/2	484/80/10	229/29/1	0.65	0.065	0.087	0.060
*TLR4*	rs1927911	−4953 C > T	Intron	199/130/26	314/218/38	142/99/17	0.48	0.256	0.258	0.258
	rs4986790	+295 A > G	Exon	321/34/0	517/53/2	241/16/1	0.34	0.048	0.050	0.035
*TLR6*	rs1039559	−502 T > C	Promoter	89/183/74	133/208/156	69/121/67	0.28	0.478	0.532	0.496
	rs5743810	+744 C > T	Exon	106/192/55	165/280/130	89/116/53	0.06	0.428	0.470	0.430
*CD14*	rs2915863	−1721 T > C	Promoter	137/154/66	191/286/96	89/135/35	0.06	0.401	0.417	0.396
	rs5744455	−651 C > T	Promoter	204/130/22	339/205/27	140/109/8	0.88	0.244	0.227	0.243
	rs2569190	−260 G > A	Promoter	107/166/83	148/286/138	64/139/54	0.24	0.466	0.491	0.481

^a^Homozygous major allele/heterozygous/homozygous minor allele; HWE: Hardy-Weinberg equilibrium; MAF: Minor allele frequency.

**Table 4:** Association between *TLR2, TLR4, TLR6* and *CD14* SNPs for genetic models with the lowest AIC and BIC scores and early- and late-asthma.

Gene	SNP	Genetic model type	Genotype	Early-onset asthma vs never asthma	Late-onset asthma vs never asthma
				OR (95% CI)	OR (95% CI)
*TLR2*	rs1898830	Additive	A allele	ref	ref
			Per G allele	0.96 (0.78–1.19)	1.12 (0.87–1.42)
	rs3804100	Dominant	TT	ref	ref
			CT/CC	1.25 (0.82–1.93)	0.88 (0.52–1.48)
	rs4969480	Dominant	AA	ref	ref
			AT/TT	1.12 (0.81–1.56)	1.17 (0.80–1.71)
*TLR4*	rs1927911	Recessive	CC/CT	ref	ref
			TT	0.80 (0.45–1.45)	0.87 (0.45–1.69)
	rs4986790	Dominant	AA	ref	ref
			AG/GG	1.23 (0.75–2.04)	0.76 (0.40–1.44)
*TLR6*	rs1039559	Dominant	CC	ref	ref
			CT/TT	0.79 (0.56–1.12)	0.78 (0.53–1.16)
	rs5743810	Dominant	TT	ref	ref
			CT/CC	0.75 (0.51–1.10)	0.73 (0.47–1.12)
*CD14*	rs2569190	Recessive	AA/AG	ref	ref
			GG	0.80 (0.58–1.10)	0.70 (0.48–1.02)
	rs5744455	Dominant	CC	ref	ref
			CT/TT	0.90 (0.67–1.21)	1.06 (0.76–1.48)
	rs2915863	Dominant	TT	ref	ref
			CT/CC	1.13 (0.77–1.67)	1.29 (0.81–2.05)

All adjusted for sex, maternal and paternal history of asthma and/or hay fever, and atopy at 7 years.

**Table 5:** Association between *TLR6* polymorphisms in an additive genetic model and childhood farm exposure on early-onset and late-onset asthma.

*TLR6* SNP	Early-onset asthma vs. never asthma					Late-onset asthma vs. never asthma				
	No childhood farm exposure	Childhood farm exposure	No childhood farm exposure	Childhood farm exposure	p-interaction between SNP and farm exposure	No childhood farm exposure	Childhood farm exposure	No childhood farm exposure	Childhood farm exposure	p-interaction between SNP and farm exposure
	n/N	n/N	OR (95% CI)	OR (95% CI)		n/N	n/N	OR (95% CI)	OR (95% CI)	
**rs1039559**										
C allele	126/483	22/53	ref	ref	0.009	58/221	4/23	ref	ref	0.38
Per T allele	357/483*	31/53*	0.79 (0.44–1.41)	0.34 (0.16–0.73)		163/221*	19/23*	0.77 (0.40–1.47)	0.68 (0.30–1.56)	
**rs5743810**										
T allele	52/311	3/26	ref	ref	0.02	102/488	21/53	ref	ref	0.52
Per C allele	259/311#	23/26#	0.77 (0.43–1.36)	0.41 (0.19–0.86)		386/488#	32/53#	0.76 (0.40–1.46)	0.77 (0.34–1.74)	

All models were adjusted for sex, maternal and paternal history of asthma and/or hay fever, and personal history of atopy at baseline.

^*^The number represents those with one or two T-alleles.

^#^The number represents those with one or two C-alleles.
