# Supplementary material for: The interaction between farming/rural environment and TLR2, TLR4, TLR6 and CD14 genetic polymorphisms in relation to early- and late-onset asthma
Source: Sci Rep. 2017 Mar 6;7:43681. doi: 10.1038/srep43681 (PMC5337969; doi:10.1038/srep43681)
Supplement: Supplementary Information [file srep43681-s1.doc]

**SUPPLEMENTARY INFORMATION**

**The interaction between farming/rural environment and *TLR2, TLR4, TLR6 and CD14* genetic polymorphisms in relation to early- and late-onset asthma**

Authors:

Melisa Y.Z. Lau, Shyamali C. Dharmage,* John A. Burgess, Aung K. Win, Adrian J. Lowe, Caroline J. Lodge, Jennifer Perret, Jennie Hui, Paul S Thomas, Stephen C. Morrison, Graham G Giles, John L. Hopper, Michael J Abramson, E Haydn Walters, Melanie C Matheson.

Table S1: The association between selected SNPs and asthma and allergy in previous studies

| SNP | Reference | Study design | Country, ethnicity | Sample size | Outcome definition | Association present | Direction of association |
| --- | --- | --- | --- | --- | --- | --- | --- |
| Gene: *CD14* | | | | | | | |
| rs2569190 | Koppelman 2001^1^ | Cross-sectional | The Netherlands, Caucasian | 317 adults | Physician diagnosed asthma | Yes | Decreased risk with CT or TT genotype |
| rs2569190 | Sharma 2004^2^ | Cross-sectional | India, Asian | 414 adults | Self-reported asthma | Yes | Decreased risk with CT or TT genotype |
| rs2569190 | Kowal 2008^3^ | Cross-sectional | Poland, Caucasian | 532 adults | Physician diagnosed asthma | Yes | Decreased risk with TT genotype |
| rs2569190 | Wang 2009^4^ | Cross-sectional | Taiwan, Asian | 956 children | Physician diagnosed asthma | Yes | Increased risk with TT genotype |
| rs5744455 | Kljaic-Bukvic 2014^5^ | Case-control | Croatia, Caucasian | 824 children | Hospital admission due to asthma | Yes | Decreased risk with T allele |
| Gene: *TLR4* | | | | | | | |
| rs4986790 and rs1927911 | Smit 2011^6^ | Cross-sectional | The Netherlands, Caucasian | 408 adults | Wheeze | No | -- |
| Gene: *TLR2* | | | | | | | |
| rs4696480 | Eder 2004^7^ | Cross-sectional | Austria and Germany, Caucasian | 609 children | Parental-reported asthma | Yes | Decreased risk with AA genotype |
| rs1898830 | Kerkhof 2010^8^ | Cohort | The Netherlands, Caucasian | 1,037 children | Doctor diagnosed asthma | No | -- |
| rs3804100 | Eder 2004^7^ | Cross-sectional | Austria and Germany, Caucasian | 609 children | Parental-reported asthma | No | -- |
| Gene: *TLR6* | | | | | | | |
| rs5743810 | Tantisira 2004^9^ | Case-control | USA, African American, European American and Hispanic American | 306 adults | Asthma | Yes | Decreased risk with T allele |
| rs5743810 | Kormann 2008^10^ | Case-control | Germany (ISAAC), Caucasian | 1,872 children | Atopic asthma | Yes | Decreased risk with CT/TT genotype |
| rs5743810 | Koponen 2014^11^ | Cohort | Finland, Caucasian | 133 children | Doctor diagnosed asthma | No | -- |
| rs1039559 | Miederma 2012^12^ | Case-control | The Netherlands, Caucasian | 192 children | Atopic disease | No | -- |

Table S2: The association between *TLR2*, *TLR4*, *TLR6* and *CD14* SNPs in a codominant, dominant, recessive and additive genetic models and early-onset and late-onset asthma

| Gene | SNP | Model | Genotype | Early-onset asthma | Late-onset asthma |
| --- | --- | --- | --- | --- | --- |
| *TLR2* | rs1898830 | Codominant | AA | ref | ref |
|  |  |  | AG | 1.05 (0.77-1.45) | 1.17 (0.81-1.67) |
|  |  |  | GG | 0.86 (0.54-1.38) | 1.21 (0.72-2.03) |
|  |  | Dominant | AA | ref | ref |
|  |  |  | AG/GG | 1.00 (0.75-1.35) | 1.17 (0.84-1.65) |
|  |  | Recessive | AA/AG | ref | ref |
|  |  |  | GG | 0.85 (0.54-1.31) | 1.12 (0.69-1.82) |
|  |  | Additive | Per G allele | 0.96 (0.78-1.19) | 1.12 (0.87-1.42) |
|  | rs3804100 | Codominant | TT | ref | ref |
|  |  |  | TC | 1.12 (0.72-1.74) | 0.88 (0.52-1.49) |
|  |  |  | CC | 4.38 (0.92-20.9) | 0.74 (0.07-8.25) |
|  |  | Dominant | TT | ref | ref |
|  |  |  | TC/CC | 1.25 (0.82-1.93) | 0.88 (0.52-1.48) |
|  |  | Recessive | TT/TC | ref | ref |
|  |  |  | CC | 4.32 (0.90-20.6) | 0.75 (0.07-8.36) |
|  |  | Additive | Per C allele | 1.33 (0.92-1.95) | 0.89 (0.56-1.43) |
|  | rs4969480 | Codominant | AA | ref | ref |
|  |  |  | AT | 1.15 (0.81-1.63) | 1.15 (0.77-1.72) |
|  |  |  | TT | 1.07 (0.71-1.61) | 1.22 (0.76-1.95) |
|  |  | Dominant | AA | ref | ref |
|  |  |  | AT/TT | 1.12 (0.81-1.56) | 1.17 (0.80-1.71) |
|  |  | Recessive | AA/AT | ref | ref |
|  |  |  | TT | 0.97 (0.69-1.38) | 1.11 (0.75-1.64) |
|  |  | Additive | Per T allele | 1.04 (0.84-1.27) | 1.11 (0.87-1.40) |
| *TLR4* | rs1927911 | Codominant | CC | ref | ref |
|  |  |  | CT | 1.06 (0.78-1.44) | 1.04 (0.73-1.48) |
|  |  |  | TT | 0.82 (0.45-1.50) | 0.89 (0.45-1.74) |
|  |  | Dominant | CC | ref | ref |
|  |  |  | CT/TT | 1.02 (0.76-1.37) | 1.01 (0.73-1.42) |
|  |  | Recessive | CC/CT | ref | ref |
|  |  |  | TT | 0.80 (0.45-1.45) | 0.87 (0.45-1.69) |
|  |  | Additive | Per T allele | 0.98 (0.77-1.24) | 0.99 (0.75-1.29) |
|  | rs4986790 | Codominant* | AA | -- | -- |
|  |  |  | AG | -- | -- |
|  |  |  | GG | -- | -- |
|  |  | Dominant | AA | ref | ref |
|  |  |  | AG/GG | 1.23 (0.75-2.04) | 0.76 (0.40-1.44) |
|  |  | Recessive* | AA/AG | -- | -- |
|  |  |  | GG | -- | -- |
|  |  | Additive | Per G allele | 1.29 (0.80-2.08) | 0.82 (0.45-1.50) |
| *TLR6* | rs1039559 | Codominant | CC | ref | ref |
|  |  |  | CT | 0.81 (0.56-1.17) | 0.75 (0.49-1.14) |
|  |  |  | TT | 0.76 (0.50-1.16) | 0.84 (0.52-1.34) |
|  |  | Dominant | CC | ref | ref |
|  |  |  | CT/TT | 0.79 (0.56-1.12) | 0.78 (0.53-1.16) |
|  |  | Recessive | CC/CT | ref | ref |
|  |  |  | TT | 0.88 (0.63-1.23) | 1.02 (0.67-1.48) |
|  |  | Additive | Per T allele | 0.88 (0.71-1.08) | 0.92 (0.73-1.17) |
|  | rs5743810 | Codominant | TT | ref | ref |
|  |  |  | TC | 0.75 (0.50-1.11) | 0.63 (0.40-1.00) |
|  |  |  | CC | 0.77 (0.49-1.18) | 0.88 (0.55-1.44) |
|  |  | Dominant | TT | ref | ref |
|  |  |  | TC/CC | 0.75 (0.51-1.10) | 0.73 (0.47-1.12) |
|  |  | Recessive | TT/TC | ref | ref |
|  |  |  | CC | 0.95 (0.69-1.31) | 1.23 (0.86-1.76) |
|  |  | Additive | Per C allele | 0.89 (0.73-1.11) | 0.99 (0.78-1.26) |
| *CD14* | rs2569190 | Codominant | AA | ref | ref |
|  |  |  | AG | 1.03 (0.71-1.49) | 1.33 (0.87-2.05) |
|  |  |  | GG | 0.81 (0.54-1.22) | 0.86 (0.53-1.39) |
|  |  | Dominant | AA | ref | ref |
|  |  |  | AG/GG | 0.94 (0.66-1.33) | 1.14 (0.76-1.71) |
|  |  | Recessive | AA/AG | ref | ref |
|  |  |  | GG | 0.80 (0.58-1.10) | 0.70 (0.48-1.02) |
|  |  | Additive | Per G allele | 0.89 (0.73-1.09) | 0.91 (0.72-1.15) |
|  | rs5744455 | Codominant | CC | ref | ref |
|  |  |  | CT | 0.95 (0.69-1.28) | 1.15 (0.81-1.62) |
|  |  |  | TT | 0.67 (0.35-1.28) | 0.54 (0.23-1.26) |
|  |  | Dominant | CC | ref | ref |
|  |  |  | CT/TT | 0.90 (0.67-1.21) | 1.06 (0.76-1.48) |
|  |  | Recessive | CC/CT | ref | ref |
|  |  |  | TT | 0.68 (0.36-1.29) | 0.51 (0.22-1.18) |
|  |  | Additive | Per T allele | 0.88 (0.69-1.13) | 0.96 (0.72-1.27) |
|  | rs2915863 | Codominant | TT | ref | ref |
|  |  |  | TC | 1.30 (0.86-1.97) | 1.51 (0.93-2.46) |
|  |  |  | CC | 0.95 (0.62-1.46) | 1.05 (0.63-1.75) |
|  |  | Dominant | TT | ref | ref |
|  |  |  | TC/CC | 1.13 (0.77-1.67) | 1.29 (0.81-2.05) |
|  |  | Recessive | TT/TC | ref | ref |
|  |  |  | CC | 0.78 (0.57-1.06) | 0.77 (0.55-1.09) |
|  |  | Additive | Per C allele | 0.92 (0.75-1.14) | 0.95 (0.75-1.21) |

*not analysed due to no observation in the GG genotype

Table S3: The degree of freedom (DF), Akaike's information criterion (AIC) and Bayesian information criterion (BIC) for each genetic model with interaction with childhood farming

| Gene | SNP | model | DF | AIC | BIC |
| --- | --- | --- | --- | --- | --- |
| *TLR2* | rs1898830 A/G | Codominant | 20 | 2106.918 | 2206.068 |
|  |  | Dominant | 16 | 2099.591 | 2178.911 |
|  |  | Recessive | 16 | 2100.17 | 2179.49 |
|  |  | **Additive** | 16 | 2099.231 | 2178.551 |
|  | rs3804100 T/C | Codominant | 18 | 2101.762 | 2191.014 |
|  |  | **Dominant** | 14 | 2094.858 | 2164.276 |
|  |  | Recessive | 16 | 2102.504 | 2181.839 |
|  |  | Additive | 16 | 2100.553 | 2179.888 |
|  | rs4969480 A/T | Codominant | 20 | 2103.637 | 2202.71 |
|  |  | **Dominant** | 16 | 2096.028 | 2175.287 |
|  |  | Recessive | 16 | 2096.745 | 2176.004 |
|  |  | Additive | 16 | 2096.258 | 2175.517 |
| *TLR4* | rs1927911 C/T | Codominant | 20 | 2094.852 | 2193.868 |
|  |  | Dominant | 16 | 2089.026 | 2168.239 |
|  |  | **Recessive** | 16 | 2087.903 | 2167.117 |
|  |  | Additive | 16 | 2089.307 | 2168.52 |
|  | rs4986790 A/G | Codominant | 20 | 2094.025 | 2193.118 |
|  |  | **Dominant** | 16 | 2091.147 | 2170.421 |
|  |  | Recessive* | -- | -- | -- |
|  |  | Additive | 16 | 2091.597 | 2170.872 |
| TLR6 | rs1039559 C/T | Codominant | 20 | 2079.826 | 2178.727 |
|  |  | **Dominant** | 16 | 2075.65 | 2154.771 |
|  |  | Recessive | 16 | 2078.306 | 2157.426 |
|  |  | Additive | 16 | 2075.288 | 2154.409 |
|  | rs5743810 T/C | Codominant | 20 | 2089.655 | 2188.767 |
|  |  | **Dominant** | 16 | 2089.617 | 2168.907 |
|  |  | Recessive | 16 | 2093.357 | 2172.646 |
|  |  | Additive | 16 | 2093.067 | 2172.356 |
| *CD14* | rs2569190 A/G | Codominant | 20 | 2094.262 | 2193.33 |
|  |  | Dominant | 16 | 2091.946 | 2171.205 |
|  |  | **Recessive** | 16 | 2089.378 | 2168.637 |
|  |  | Additive | 16 | 2091.348 | 2170.607 |
|  | rs5744455 C/T | Codominant | 20 | 2096.215 | 2195.27 |
|  |  | **Dominant** | 16 | 2090.327 | 2169.571 |
|  |  | Recessive | 16 | 2092.059 | 2171.302 |
|  |  | Additive | 16 | 2090.772 | 2170.015 |
|  | rs2915863 T/C | Codominant | 20 | 2100.362 | 2199.474 |
|  |  | **Dominant** | 16 | 2096.84 | 2176.13 |
|  |  | Recessive | 16 | 2097.093 | 2176.383 |
|  |  | Additive | 16 | 2098.333 | 2177.623 |

All models were adjusted for sex, maternal and paternal history of asthma and/or hay fever, and atopy at 7 years;

*not analysed due no observation in the GG genotype

Table S4: The interaction between *TLR6* SNPs in codominant, dominant and recessive genetic models and childhood farm exposure for early-onset and late-onset asthma

|  |  |  |  | **Early-onset asthma vs. never asthma** | | | | **Late-onset asthma vs. never asthma** | | | |
| --- | --- | --- | --- | --- | --- | --- | --- | --- | --- | --- | --- |
| Gene | SNP | Model | Genotype | No childhood farm exposure |  | Childhood farm exposure | p-interaction between SNP and farm exposure | No childhood farm exposure |  | Childhood farm exposure | p-interaction between SNP and farm exposure |
|  |  |  |  | OR (95% CI) |  | OR (95% CI) |  | OR (95% CI) |  | OR (95% CI) |  |
| *TLR6* | rs1039559 | Codominant | CC | ref |  | ref |  | ref |  | ref |  |
|  |  |  | CT | 0.83 (0.36-1.92) |  | 0.30 (0.07-1.34) | 0.16 | 0.55 (0.23-1.32) |  | 1.17 (0.19-7.17) | 0.65 |
|  |  |  | TT | 2.12 (0.82-5.52) |  | 0.13 (0.03-0.60) | 0.01 | 1.49 (0.55-4.05) |  | 0.56 (0.09-3.61) | 0.55 |
|  |  | Dominant | CC | ref |  | ref |  | ref |  | ref |  |
|  |  |  | CT/TT | 1.25 (0.67-2.32) |  | 0.21 (0.05-0.83) | 0.04 | 0.84 (0.44-1.61) |  | 0.84 (0.15-4.67) | 0.99 |
|  |  | Recessive | CC/CT | ref |  | ref |  | ref |  | ref |  |
|  |  |  | TT | 0.56 (0.28-1.10) |  | 0.28 (0.9-0.84) | 0.03 | 0.62 (0.28-1.34) |  | 0.49 (0.15-1.64) | 0.17 |
|  |  |  |  |  |  |  |  |  |  |  |  |
|  | rs5743810 | Codominant | TT | ref |  | ref |  | ref |  | ref |  |
|  |  |  | CT | 0.90 (0.39-2.10) |  | 0.28 (0.06-1.28) | 0.17 | 0.43 (0.18-1.03) |  | 1.93 (0.27-13.9) | 0.26 |
|  |  |  | CC | 1.65 (0.67-4.06) |  | 0.17 (0.04-0.81) | 0.04 | 1.59 (0.59-4.27) |  | 0.88 (0.12-6.63) | 0.89 |
|  |  | Dominant | TT | ref |  | ref |  | ref |  | ref |  |
|  |  |  | CT/CC | 1.19 (0.64-2.18) |  | 0.22 (0.06-0.90) | 0.06 | 0.76 (0.40-1.44) |  | 1.35 (0.20-8.99) | 0.54 |
|  |  | Recessive | TT/CT | ref |  | ref |  | ref |  | ref |  |
|  |  |  | CC | 0.59 (0.30-1.18) |  | 0.39 (0.13-1.16) | 0.08 | 0.56 (0.26-1.23) |  | 0.51 (0.15-1.68) | 0.11 |

All models were adjusted for sex, maternal and paternal history of asthma and/or hay fever, and atopy at 7 years;

Table S5: The interaction between *TLR2*, *TLR4* and *CD14* SNPs for genetic models with the lowest AIC and BIC scores and childhood farm exposure for early- and late-onset asthma

|  |  |  |  | **Early-onset asthma vs. never asthma** | | | | **Late-onset asthma vs. never asthma** | | | |
| --- | --- | --- | --- | --- | --- | --- | --- | --- | --- | --- | --- |
| Gene | SNP | Model | Genotype | No childhood farm exposure |  | Childhood farm exposure | p-interaction between SNP and farm exposure | No childhood farm exposure |  | Childhood farm exposure | p-interaction between SNP and farm exposure |
|  |  |  |  | OR (95% CI) |  | OR (95% CI) |  | OR (95% CI) |  | OR (95% CI) |  |
| *TLR2* | rs1898830 | Additive | A allele | ref |  | ref |  | ref |  | ref |  |
|  |  |  | Per G allele | 0.82 (0.44-1.49) |  | 1.04 (0.48-2.26) | 0.843 | 0.92 (0.46-1.84) |  | 0.83 (0.34-1.98) | 0.490 |
|  | rs3804100 | Dominant | TT | ref |  | ref |  | ref |  | ref |  |
|  |  |  | CT/CC | 1.35 (0.79-2.26) |  | 1.16 (0.25-5.32) | 0.900 | 1.06 (0.57-1.96) |  | 1.05 (0.15-7.22) | 0.869 |
|  | rs4969480 | Dominant | AA | ref |  | ref |  | ref |  | ref |  |
|  |  |  | AT/TT | 0.84 (0.45-1.55) |  | 1.15 (0.35-3.79) | 0.997 | 0.87 (0.43-1.76) |  | 0.96 (0.26-3.61) | 0.729 |
| *TLR4* | rs1927911 | Recessive | CC/CT | ref |  | ref |  | ref |  | ref |  |
|  |  |  | TT | 0.26 (0.02-3.11) |  | 2.62 (0.23-30.3) | 0.327 | 0.63 (0.04-11.0) |  | 1.13 (0.07-19.4) | 0.849 |
|  | rs4986790 | Dominant | AA | ref |  | ref |  | ref |  | ref |  |
|  |  |  | AG/GG | 1.32 (0.79-2.19) |  | 0.87 (0.17-4.43) | 0.642 | 0.50 (0.23-1.04) |  | 1.15 (0.20-6.47) | 0.636 |
| *CD14* | rs2569190 | Recessive | AA/AG | ref |  | ref |  | ref |  | ref |  |
|  |  |  | GG | 0.63 (0.24-1.68) |  | 1.11 (0.36-3.39) | 0.524 | 1.10 (0.32-3.85) |  | 0.51 (0.13-2.01) | 0.605 |
|  | rs5744455 | Dominant | CC | ref |  | ref |  | ref |  | ref |  |
|  |  |  | CT/TT | 0.89 (0.63-1.26) |  | 2.16 (0.58-8.00) | 0.595 | 1.01 (0.68-1.48) |  | 2.89 (0.79-10.6) | 0.241 |
|  | rs2915863 | Dominant | TT | ref |  | ref |  | ref |  | ref |  |
|  |  |  | CT/CC | 0.73 (0.39-1.32) |  | 2.26 (0.42-8.22) | 0.272 | 0.83 (0.42-1.65) |  | 1.15 (0.29-4.49) | 0.823 |

All models were adjusted for sex, maternal and paternal history of asthma and/or hay fever, and atopy at 7 years;

Table S6: Association between *TLR6* polymorphisms in an additive genetic model and early-onset transient and early-onset persistent asthma by childhood farm exposure

| *TLR6* SNP | **Early-onset (transient) vs. never asthma** | | | | |  | **Early-onset (persistent) vs. never asthma** | | | | |
| --- | --- | --- | --- | --- | --- | --- | --- | --- | --- | --- | --- |
|  | No childhood farm exposure | Childhood farm exposure | No childhood farm exposure | Childhood farm exposure | p-interaction between SNP and farm exposure |  | No childhood farm exposure | Childhood farm exposure | No childhood farm exposure | Childhood farm exposure | p-interaction between SNP and farm exposure |
|  | n/N | n/N | OR (95% CI) | OR (95% CI) |  |  | n/N | n/N | OR (95% CI) | OR (95% CI) |  |
| rs1039559 |  |  |  |  |  |  |  |  |  |  |  |
| C allele | 40/155 | 11/25 | ref | ref |  |  | 86/328 | 11/28 | ref | ref |  |
| Per T allele | 115/155* | 14/25* | 0.54 (0.28-1.04) | 0.32 (0.13-0.74) | 0.01 |  | 242/328* | 17/28* | 1.09 (0.56-2.11) | 0.39 (0.17-0.92) | 0.04 |
| rs5743810 |  |  |  |  |  |  |  |  |  |  |  |
| T allele | 32/154 | 10/25 | ref | ref |  |  | 70/334 | 11/28 | ref | ref |  |
| Per C allele | 122/154# | 15/25# | 0.50 (0.26-1.00) | 0.41 (0.18-0.95) | 0.05 |  | 264/334# | 17/28# | 1.10 (0.57-2.13) | 0.41 (0.18-0.97) | 0.05 |

All models were adjusted for sex, maternal and paternal history of asthma and/or hay fever, and atopy at 7 years;

*the number represents those with one or two T alleles

# the number represents those with one or two C alleles

Table S7: Association between *TLR6* polymorphisms in an additive genetic model and asthma by childhood farm exposure after removing children who has asthma before or at age 5 years

| *TLR6* SNP | **Early-onset asthma vs never asthma** | | | | |  | **Late-onset asthma vs never asthma** | | | | |
| --- | --- | --- | --- | --- | --- | --- | --- | --- | --- | --- | --- |
|  | No childhood farm exposure | Childhood farm exposure | No childhood farm exposure | Childhood farm exposure | p-interaction between SNP and farm exposure |  | No childhood farm exposure | Childhood farm exposure | No childhood farm exposure | Childhood farm exposure | p-interaction between SNP and farm exposure |
|  | n/N | n/N | OR (95% CI) | OR (95% CI) |  |  | n/N | n/N | OR (95% CI) | OR (95% CI) |  |
| rs1039559 |  |  |  |  |  |  |  |  |  |  |  |
| C allele | 24/117 | 6/10 | ref | ref |  |  | 43/159 | 4/19 | ref | ref |  |
| Per T allele | 93/117* | 4/10* | 0.54 (0.28-1.04) | 0.32 (0.13-0.74) | 0.01 |  | 116/159* | 15/19* | 1.09 (0.56-2.11) | 0.39 (0.17-0.92) | 0.04 |
| rs5743810 |  |  |  |  |  |  |  |  |  |  |  |
| T allele | 18/115 | 6/10 | ref | ref |  |  | 33/160 | 3/19 | ref | ref |  |
| Per C allele | 97/115# | 4/10# | 0.50 (0.26-1.00) | 0.41 (0.18-0.95) | 0.05 |  | 127/160# | 16/19# | 1.10 (0.57-2.13) | 0.41 (0.18-0.97) | 0.05 |

All models were adjusted for sex, maternal and paternal history of asthma and/or hay fever, and atopy at 7 years;

*the number represents those with one or two T alleles

# the number represents those with one or two C alleles

Reference

1 Koppelman, G. H. *et al.* Association of a promoter polymorphism of the CD14 gene and atopy. *American journal of respiratory and critical care medicine* **163**, 965-969, doi:10.1164/ajrccm.163.4.2004164 (2001).

2 Sharma, M. *et al.* Suggestive evidence of association of C-159T functional polymorphism of the CD14 gene with atopic asthma in northern and northwestern Indian populations. *Immunogenetics* **56**, 544-547, doi:10.1007/s00251-004-0721-y (2004).

3 Kowal, K. *et al.* Analysis of -675 4 g/5 G SERPINE1 and C-159T CD14 polymorphisms in house dust mite-allergic asthma patients. *Journal of investigational allergology & clinical immunology* **18**, 284-292 (2008).

4 Wang, J. Y., Liou, Y. H., Wu, Y. J., Hsiao, Y. H. & Wu, L. S. An association study of 13 SNPs from seven candidate genes with pediatric asthma and a preliminary study for genetic testing by multiple variants in Taiwanese population. *Journal of clinical immunology* **29**, 205-209, doi:10.1007/s10875-008-9256-6 (2009).

5 Kljaic-Bukvic, B. *et al.* Genetic variants in endotoxin signalling pathway, domestic endotoxin exposure and asthma exacerbations. *Pediatric allergy and immunology : official publication of the European Society of Pediatric Allergy and Immunology* **25**, 552-557, doi:10.1111/pai.12258 (2014).

6 Smit, L. A. *et al.* Endotoxin exposure, CD14 and wheeze among farmers: a gene--environment interaction. *Occupational and environmental medicine* **68**, 826-831, doi:10.1136/oem.2010.060038 (2011).

7 Eder, W. *et al.* Toll-like receptor 2 as a major gene for asthma in children of European farmers. *The Journal of allergy and clinical immunology* **113**, 482-488, doi:10.1016/j.jaci.2003.12.374 (2004).

8 Kerkhof, M. *et al.* Toll-like receptor 2 and 4 genes influence susceptibility to adverse effects of traffic-related air pollution on childhood asthma. *Thorax* **65**, 690-697, doi:10.1136/thx.2009.119636 (2010).

9 Tantisira, K. *et al.* Toll-like receptor 6 gene (TLR6): single-nucleotide polymorphism frequencies and preliminary association with the diagnosis of asthma. *Genes and immunity* **5**, 343-346, doi:10.1038/sj.gene.6364096 (2004).

10 Kormann, M. S. *et al.* Toll-like receptor heterodimer variants protect from childhood asthma. *The Journal of allergy and clinical immunology* **122**, 86-92, 92 e81-88, doi:10.1016/j.jaci.2008.04.039 (2008).

11 Koponen, P. *et al.* The association of genetic variants in toll-like receptor 2 subfamily with allergy and asthma after hospitalization for bronchiolitis in infancy. *The Pediatric infectious disease journal* **33**, 463-466, doi:10.1097/INF.0000000000000253 (2014).

12 Miedema, K. G. *et al.* Polymorphisms in the TLR6 gene associated with the inverse association between childhood acute lymphoblastic leukemia and atopic disease. *Leukemia* **26**, 1203-1210, doi:10.1038/leu.2011.341 (2012).
